# Gut-lung immunometabolic crosstalk in sepsis: from microbiota to respiratory failure

**DOI:** 10.3389/fmed.2025.1685044

**Published:** 2025-10-30

**Authors:** Qixiu Li, Xi-Cheng Song, Kefeng Li, Jing Wang

**Affiliations:** ^1^The Second Clinical Medical College of Binzhou Medical University, Yantai, China; ^2^Department of Critical Care Medicine, Yantai Yuhuangding Hospital, Qingdao University, Yantai, China; ^3^Department of Otolaryngology Head and Neck Surgery, Yantai Yuhuangding Hospital, Qingdao University, Yantai, China; ^4^Faculty of Applied Sciences, Macao Polytechnic University, Macao, Macao SAR, China

**Keywords:** sepsis, gut-lung axis, microbiota dysbiosis, intensive care unit, immunometabolism

## Abstract

Sepsis is a systemic immune-metabolic disorder syndrome caused by infection, in which gut microbiota dysbiosis plays a central role in the occurrence and development of multi-organ dysfunction. This paper systematically elaborates on the bidirectional regulatory mechanism of the “gut-lung axis” in sepsis. Gut microbiota dysregulation damages the gut barrier function, reduces the production of short-chain fatty acids (SCFAs), and increases endotoxin translocation. Subsequently, it activates alveolar macrophage polarization, promotes the formation of neutrophil extracellular traps (NETs), and leads to an imbalance in the Treg/Th17 cell ratio, ultimately exacerbating the pathological process of acute lung injury (ALI) or acute respiratory distress syndrome (ARDS). Conversely, the pulmonary inflammatory response can also aggravate gut barrier damage through circulating inflammatory mediators, forming a vicious cycle. Mechanistically, HIF-1α, mTOR, and Sirtuins do not act in isolation. Instead, they jointly regulate the metabolic fate of immune cells through spatiotemporally dynamic interactions. During the evolution of sepsis, these signals exhibit opposite regulatory polarities during the hyper-inflammatory phase and the immunosuppressive phase, and mitochondrial dysfunction and oxidative stress further amplify the inflammatory cascade reaction. Preclinical research evidence shows that microbiota-based intervention measures (including probiotic preparations, fecal microbiota transplantation, and SCFA supplementation) and vagus nerve electrical stimulation can effectively alleviate sepsis-related lung injury and improve prognosis, but there is significant individual heterogeneity in their therapeutic effects. Future research should not be restricted to descriptive associations. Instead, it is essential to conduct in-depth analyses of the specific logic of the aforementioned signaling networks in terms of cell types, subcellular compartments, and disease course timings, and clarify their context-dependent controversies to promote the transformation of mechanistic understanding into precision treatment. Meanwhile, research efforts should focus on constructing a multi-omics dynamic biomarker system integrating metagenomics, metabolomics, and immunophenotyping analysis and designing clinical trials through precise patient stratification to facilitate the clinical translation of individualized treatment strategies based on gut-lung axis regulation.

## 1 Introduction

Sepsis is a condition in which infection triggers a dysregulated host immune response, leading to multiple organ dysfunction through complex pathophysiological mechanisms (1). Epidemiological data indicate that the mortality rate of sepsis is 15–25% in high-income countries, while it exceeds 40% in middle-and low-income countries (2). Globally, there are approximately 50 million new cases and 11 million deaths annually, making sepsis one of the leading causes of death in critically ill patients (2). Currently, its pathological mechanisms, especially the effects on remote organ damage, have not been fully elucidated.

The latest research shows that the gut microbiota plays a crucial role in systemic inflammation and metabolic homeostasis through the “gut-lung axis” regulatory network (including mechanisms such as microbial migration, immune regulation, and metabolite exchange) (3). However, the sepsis molecular mechanisms still need in-depth study. During the pathological process of sepsis, the systemic inflammatory response triggered by infection and the widespread use of antibiotics jointly lead to imbalances in the gut and lung microecologies (4–6). This dysbiosis not only impairs the intestinal barrier function, increasing the risk of enterogenic infections but also disrupts the pulmonary immune microenvironment through blood circulation, lymphatic return, and neuroendocrine pathways, exacerbating lung injury (7). Notably, pulmonary inflammation can also inversely regulate the gut microenvironment (8), thus forming a vicious cycle of bidirectional gut-lung regulation, which significantly increases the risk of poor prognosis in patients.

Despite the continuous improvement of supportive treatment methods, the current clinical management of sepsis remains predominantly reactive, lacking targeted intervention strategies that can effectively interrupt the vicious cycle of organ dysfunction (9, 10). Therefore, there is an urgent need to drive a fundamental transformation of treatment concepts. This review proposes and demonstrates the following core idea: Immune-metabolic dysregulation is the key pathophysiological mechanism driving the vicious cycle of the gut-lung axis in sepsis. It has been found that microbial metabolites from the gut are not only regulators of inflammation but also the “initial instructions” for reprogramming the energy metabolic pathways of lung immune cells (such as macrophages and neutrophils). This metabolic reprogramming, characterized by abnormal activation of glycolysis and mitochondrial dysfunction, ultimately determines the fate and function of immune cells, leading to the occurrence and development of lung injury.

Based on the above-mentioned viewpoints, this paper will systematically elaborate on the mechanisms by which sepsis induces gut microbiota dysbiosis and the imbalance of its metabolite homeostasis, and focus on analyzing how key signaling pathways such as hypoxia-inducible factor-1α (HIF-1α) and mammalian target of rapamycin (mTOR) mediate the metabolic and functional abnormalities of lung immune cells. This study will go beyond traditional descriptive associations, critically evaluate the limitations of existing treatment strategies from a metabolic perspective, and further propose a precision patient-typing framework based on biomarkers. Ultimately, it is expected to shift the treatment concept of sepsis from “one-size-fits-all immunosuppression” or “blind probiotic intervention” to “precision immune-metabolic editing,” providing new directions for future research and practice.

## 2 Methods

This narrative review systematically retrieved studies related to sepsis, critical illness, the gut-lung axis, and microbiota from the establishment of the PubMed, web of science, and Scopus database to August 2025. The retrieval strategy comprehensively used the following keywords: sepsis, critically ill patients, gut-lung axis, microbiota, acute lung injury, acute respiratory distress syndrome, bile acids, tryptophan, cholinergic anti-inflammatory pathway, HIF-1α, mTOR, Sirtuins, macrophage polarization, NETs, Toll-like receptors (TLR), oxidative stress, short-chain fatty acids, fecal microbiota transplantation, probiotics, prebiotics, dietary fiber, inulin, fructooligosaccharides, etc. The types of included literature were clinical trials, animal experiments, *in vitro* studies, reviews, and meta-analyses. The primary criteria for literature screening were the research question and clinical relevance. High-quality literature that was scientifically rigorous, had a clear mechanism, and was recently published was preferentially selected. The studies finally included should focus on the gut-lung axis mechanism or relevant intervention strategies in sepsis, ALI/ARDS. Conference abstracts, dissertations, studies irrelevant to the topic, those with incomplete data, and duplicate publications were excluded. In addition, to avoid missing key mechanistic studies, supplementary searches were conducted on the references of the included studies, in addition to the retrieval of relevant keywords. Given that this is a narrative review, no formal quality assessment tools were used. However, the credibility and reliability of the included studies were considered during the data synthesis and interpretation process.

## 3 Changes in the gut microbiome induced by sepsis

The human gut microbiota constitutes a highly complex microecosystem, whose composition includes various microbial groups such as bacteria, fungi, and viruses. In a healthy gut microbiota, beneficial bacteria genera such as Bifidobacterium and Lactobacillus are the dominant populations, which play important physiological roles by maintaining the integrity of the gut barrier, promoting the metabolism and absorption of nutrients, and regulating the host’s immune homeostasis (11). However, factors such as changes in diet structure, exposure to environmental factors, and changes in lifestyle can disrupt this microecological balance, leading to dysbiosis in the composition and function of the microbiota (12, 13). A large-scale observational cohort study confirmed that gut microbiota dysbiosis in ICU patients was significantly positively correlated with prolonged hospital stay and increased all-cause mortality. Sepsis patients exhibit a characteristic pattern of dual-system dysregulation of the “immune-microbiome” (14), with neutrophilia and a pro-inflammatory cytokine storm in the early stage, and lymphopenia and immune paralysis in the later stage (14). This immune dynamic change interacts with gut microbiota dysbiosis, forming a vicious cycle of “microbiota dysbiosis-immune dysregulation,” which further exacerbates the progression of the disease.

### 3.1 Pathological changes in gut microbiota composition

Sepsis leads to characteristic pathological changes in the gut microecosystem, and the mechanism involves the synergistic action of multiple factors. Histopathological evidence shows that approximately 50% of ICU patients have significant damage to the intestinal epithelial structure (15). During shock resuscitation, catecholamine drugs not only induce intestinal ischemia-reperfusion injury but also damage the integrity of the mucus barrier through microcirculatory disorders, aggravated mucosal inflammation, and inhibition of crypt cell proliferation (16). In addition, the gut microecosystem of sepsis patients is also faced with multiple iatrogenic damaging factors, including the systemic inflammatory response, changes in enteral nutrition formulas, loss of the gastric acid barrier caused by proton-pump inhibitors, and the non-discriminatory killing effect of broad-spectrum antibiotics. These factors together contribute to the “ICU microbiome crisis” (17). Clinical studies have confirmed that the *α*-diversity (Shannon index and Chao1 index) of the gut microbiota in sepsis patients is significantly negatively correlated with the severity of the disease (18). As the condition worsens, patients present with a pattern of microbiota imbalance characterized by a decrease in the abundance of beneficial bacteria (Bifidobacterium, Lactobacillus) and the proliferation of pathogenic bacteria (*Escherichia coli*, *Klebsiella pneumoniae*) (19).

In-depth mechanism studies in mouse models have shown that the over-proliferation of pathogens exacerbates the progression of the disease through multiple pathways. The endotoxins released by pathogens can activate the TLR4/NF-κB signaling pathway, leading to a significant increase in the levels of pro-inflammatory cytokines (IL-6 and TNF-α) (20). At the same time, the enterotoxins secreted by pathogens can damage the tight-junction structure of the intestinal epithelium, significantly increasing the risk of bacterial translocation (21, 22). Longitudinal clinical monitoring data show that for every one-log increase in the relative abundance of Enterobacteriaceae within 7 days after admission to the ICU, the 180-day death risk of patients increases by 92% (23).

### 3.2 Microbial metabolite transfer: consumption of short-chain fatty acids and toxin accumulation

SCFAs, as the core products of the gut microbiota-dietary fiber metabolism (24–26), have significantly reduced levels in sepsis patients and are negatively correlated with the severity of the disease (19). This metabolic abnormality results from the combined effects of a decrease in the abundance of SCFA-producing bacteria (Bifidobacterium), impaired intestinal mucosal absorption function, and insufficient dietary fiber intake. The lack of SCFAs exacerbates the deterioration of sepsis through multiple pathological mechanisms: the resulting energy metabolic disorder of intestinal epithelial cells can down-regulate the expression of junction proteins (ZO-1), damage the gut barrier function, and promote endotoxin translocation; in the early stage of the disease, the reduction of SCFAs weakens the regulatory balance of Treg/Th17 cells, exacerbating the pro-inflammatory response; in the later stage, it aggravates the immunosuppressive state due to immune regulatory dysfunction, ultimately forming a vicious cycle in which microbiota dysbiosis and immune dysregulation exacerbate each other (27). It is worth noting that SCFAs and secondary bile acid metabolism are synergistic, and they jointly affect the prognosis of sepsis, providing a new therapeutic target for metabolic intervention (19).

In contrast to the consumption of SCFAs, gut toxins in sepsis patients accumulate significantly, mainly manifested as a significant increase in lipopolysaccharide (LPS) in the cell walls of Gram-negative bacteria and protein exotoxins secreted by bacteria (*Clostridium perfringens* alpha-toxin) (28, 29). The proliferation of Gram-negative bacteria can promote the release of LPS, which activates intestinal immune cells to trigger a local inflammatory response and spreads through the bloodstream, leading to multi-organ dysfunction syndrome (30, 31); at the same time, exotoxins secreted by pathogens (*Staphylococcus aureus* alpha-toxin) directly cause tissue damage (32). The accumulation of these toxic substances not only directly damages the gut barrier function but also induces systemic inflammatory response syndrome by over-activating the immune system, forming a vicious cycle of “toxin accumulation-inflammation exacerbation.” It is worth noting that such toxins can also interfere with the neuroendocrine regulation of the gut, aggravating gastrointestinal dysfunction and disrupting microbiota homeostasis. Therefore, clarifying the interaction mechanism between gut toxin accumulation and immune dynamic changes is of great theoretical significance for the targeted treatment of sepsis.

The above-mentioned mechanism of short-chain fatty acids (SCFAs) depletion not only reveals the pathological process of sepsis deterioration but also shows clear clinical translation potential. In terms of diagnosis, the detection of fecal butyric acid concentration or the plasma SCFAs profile is expected to become an objective biomarker for evaluating the disease severity and predicting the 28-day mortality, providing a new basis for early risk stratification (33). At the treatment level, although the supplementation of sodium butyrate has shown gut-protective effects in preclinical studies, its clinical translation still faces many challenges, including the selection of delivery routes (weighing the advantages and disadvantages of oral, enema, or intravenous administration), the determination of the intervention time window (the difference in efficacy between the inflammatory storm period and the immunosuppressive period), and the non-targeted effects that may be caused by systemic administration (34–36). These challenges suggest that future intervention strategies should focus more on promoting the endogenous production of SCFAs through precisely formulated soluble dietary fiber or developing butyric acid precursor drugs for colon-targeted delivery, so as to improve the efficacy and ensure the clinical safety of treatment.

## 4 Disorders of immune metabolic pathways in sepsis

### 4.1 Key components and mechanisms of lung immunity

The lung is the only internal organ in the human body that is in direct contact with the external environment continuously (17). Every day, a large amount of air that may carry bacteria, viruses, and allergens is inhaled through the respiratory tract (37). Microbiome analysis based on sputum, lung tissue, and bronchoalveolar lavage fluid has confirmed that the lungs of healthy individuals have a complex microbial ecosystem characterized by low-abundance Firmicutes, Bacteroidetes, and Proteobacteria (38). This unique immune microenvironment is crucial for maintaining health.

Alveolar macrophages (AMs) mainly rely on mitochondrial oxidative phosphorylation (OXPHOS) in the resting state, but during sepsis (39), they switch to glycolysis through HIF-1α-mediated metabolic reprogramming, resulting in excessive secretion of pro-inflammatory cytokines (40). In the acute inflammatory phase, pulmonary neutrophils clear pathogens and regulate the immune response by releasing antibacterial effector molecules and pro-inflammatory cytokines, but over-activation can cause tissue damage (41). Studies on sepsis mouse models have shown that up-regulation of PD-L1 can inhibit the activity of caspase-3 and up-regulate the expression of Mcl-1 through the PD-1/PI3K/Akt signaling pathway, thereby delaying neutrophil apoptosis, leading to their abnormal accumulation in the lungs and exacerbating lung injury (42). In addition, the destruction of the tight-junction protein (Claudin-4) in alveolar epithelial cells can lead to bacterial translocation and increased infiltration of inflammatory cells, exacerbating the inflammatory response (43).

### 4.2 Excessive inflammation and immunosuppression: metabolic reprogramming of immune cells

During the progression of sepsis, the immunometabolic state exhibits highly dynamic changes, characterized by a transition from an early hyper-inflammatory state to a late persistent immunosuppressive state. The core mechanism of this transition lies in the profound metabolic reprogramming of immune cells.

In the early hyper-inflammatory stage, innate immune cells (such as macrophages and neutrophils) undergo a rapid switch from oxidative phosphorylation to glycolysis (44). Hypoxia and inflammatory signals (such as LPS and succinate) can stabilize HIF-1α, which in turn upregulates the expression of glycolytic enzymes and pro-inflammatory genes (such as IL-1β) at the transcriptional level (45). Meanwhile, the activation of mammalian target of rapamycin complex 1 (mTORC1) promotes protein synthesis and inhibits autophagy, further enhancing the glycolytic and pro-inflammatory phenotypes (46). This metabolic remodeling, similar to the “Warburg effect,” can rapidly produce adenosine triphosphate (ATP) and biosynthetic precursors to meet the energy requirements for cytokine production and phagocytosis (47). However, continuous glycolytic flux leads to lactate accumulation. Lactate stabilizes HIF-1α through a positive feedback loop, which instead exacerbates the inflammatory response and causes acidotic tissue damage (48, 49).

As the disease progresses to the late immunosuppressive stage, significantly limited energy metabolism becomes a prominent feature. Mitochondrial dysfunction is the core aspect of this stage, mainly manifested as a decrease in mitochondrial membrane potential (reversible collapse occurs in some cells), reduced ATP synthesis efficiency, and abnormal release of reactive oxygen species (ROS) and mitochondrial DNA (mtDNA) (50, 51). Meanwhile, the activity of nicotinamide adenine dinucleotide (NAD^+^)-dependent deacetylases Sirtuins [especially sirtuin 1 (SIRT1) and sirtuin 3 (SIRT3)] decreases due to NAD^+^ depletion, leading to hyperacetylation of multiple target proteins, which tends to enhance NF-κB signaling and inhibit fatty acid oxidation (52, 53). The mTOR signaling pathway plays a dual role in a time-dependent manner during the progression of sepsis: in the early stage, its activation promotes the activation of the NLR family pyrin domain-containing 3 (NLRP3) inflammasome, mediating the “cytokine storm”; while its continuous activation in T cells induces pyroptosis by inhibiting the peroxisome proliferator-activated receptor *γ* (PPARγ)-nuclear factor erythroid 2-related factor 2 (Nrf2) axis, leading to immunosuppression (54).

Currently, there are still significant controversies and gaps in the understanding of these pathway mechanisms. The role of HIF-1α is particularly context-dependent: although it is recognized as an inflammation driver, some studies suggest that it may play a protective role by enhancing antibacterial defense in specific microenvironments (such as the early stage of bacterial infection in the epithelial barrier) (55). The controversial outcomes of mTOR inhibitors in sepsis clinical trials highlights the extreme importance of the intervention timing and immune-metabolic stratification of patients-inhibiting mTOR during the immunosuppressive stage may further impair T-cell function and exacerbate the condition (56). In addition, Sirtuins and HIF-1α form a key metabolic-inflammatory regulatory node, but their cell-type specificity and dynamic range still need to be systematically analyzed (57, 58).

Future research must integrate single-cell spatiotemporal omics and dynamic metabolic markers. Based on the dynamic analysis of the specific roles and interactions of key molecules such as HIF-1α and mTOR in different stages and cell types during sepsis, the individualized treatment window should be accurately defined, and then the targeted intervention of the immune-metabolic network should be realized from “empirical” to “precision”-that is, specifically inhibiting inflammatory metabolism in the early hyper-inflammatory stage and effectively repairing cellular energy exhaustion in the late immunosuppressive stage to avoid exacerbating the condition due to incorrect medication.

### 4.3 Mitochondrial dysfunction and oxidative stress in gut-lung crosstalk

Mitochondria, as the core hub connecting gut-derived signals and pulmonary immunometabolism, play a crucial role in the pathogenesis of sepsis. During sepsis, the gut barrier is disrupted, allowing microbial products (such as endotoxins) to enter the circulatory system, which can directly damage the mitochondrial function of distant organs (including the lungs). This mitochondrial damage is not merely a consequence of inflammation but a key amplifier of the vicious cycle in the gut-lung axis.

The oxidative phosphorylation capacity of damaged mitochondria significantly declines, leading to an energy crisis in immune cells and epithelial cells. More importantly, damaged mitochondria leak excessive reactive oxygen species (ROS) and mtDNA. Among them, mtDNA, as an effective damage-associated molecular pattern (DAMPs), can activate the NLRP3 inflammasome in the cytoplasm, promoting the release of IL-1β and IL-18 (59). Meanwhile, mtDNA can also activate the production of type I interferons through the cyclic guanosine monophosphate-adenosine monophosphate synthase (cGAS)-stimulator of interferon genes (STING) pathway (60). These reactions form a feed-forward loop, where inflammation triggers more mitochondrial damage, and mitochondrial damage in turn exacerbates the inflammatory response.

The fate of mitochondria is precisely regulated by mitophagy, and this process is closely linked to the core immunometabolic pathways. During the high-inflammation phase, moderate mitophagy can timely clear damaged mitochondria and limit the release of ROS and mtDNA, thereby exerting a cytoprotective effect (61). However, during the immunosuppressive phase, mitophagy may become excessive or dysregulated, leading to the premature clearance of still-functional mitochondria, which in turn affects cellular energy metabolism and immune function (62). This regulatory mechanism involves key protein pathways such as PTEN-induced putative kinase 1 (PINK1)/Parkin, and its activity is regulated by the Sirtuins family and the mammalian target of rapamycin (mTOR) signaling pathway, further reflecting the multi-level and inter-related regulatory characteristics of the immunometabolic network (63–65).

Therefore, targeted regulation of mitochondrial quality control, timely enhancing mitophagy in the early stage of the disease or effectively curbing its over-activation in the late stage, is a promising but challenging treatment strategy. The successful application of this strategy depends on the precise discrimination of the disease stage. Future research needs to delve from macroscopic associations into microscopic mechanisms. The core tasks are to elucidate the molecular program by which gut signals initiate lung mitochondrial damage and the critical transition nodes at which mitophagy shifts from being protective to destructive during the disease process. The ultimate goal of the research is to develop mitochondrial-targeted therapies that can intelligently respond to pathological states and to achieve stage-based individualized diagnosis and treatment of sepsis using mitochondrial function markers, thereby systematically innovating the existing clinical prevention and treatment strategies.

### 4.4 Key pathways in sepsis-associated organ failure: HIF-1*α*, mTOR, and Sirtuins

#### 4.4.1 HIF-1α pathway

HIF-1α forms a heterodimer by binding the α-subunit with the β-subunit (usually ARNT) to exert transcriptional regulatory functions (66, 67). Under normoxic conditions, prolyl hydroxylase (PHD)-mediated hydroxylation of HIF-1α promotes VHL-dependent ubiquitin-proteasome degradation (68). In hypoxia, PHD activity is inhibited, and HIF-1α escapes degradation due to reduced hydroxylation, leading to a significant accumulation of its protein level (69, 70). Additionally, signaling pathways such as PI3K/Akt/mTOR and metabolites like succinate can also regulate the expression and stability of HIF-1α (71, 72). Specifically, succinate can inhibit the activity of PHD, block the prolyl hydroxylation of HIF-1α, and then inhibit the pVHL-mediated ubiquitination degradation process (73). This mechanism enhances the stability of the HIF-1α protein and ultimately upregulates the transcription and expression of downstream target genes (such as IL-1β) in a HIF-1*α*-dependent manner (74).

In sepsis, bacterial infection induces inflammatory hypoxia by increasing cellular oxygen consumption and immune cell infiltration (75). Bacterial components such as LPS stabilize HIF-1α by activating the MAPK/NF-κB pathway (76), and pro-inflammatory cytokines such as IL-6 and TNF-α released by immune cells further upregulate HIF-1α expression (77, 78). In viral sepsis, HIF-1α accumulation mainly depends on the combined action of viral proteins (e.g., C16 of VACV or LMP1 of Epstein-Barr virus) on PHD activity regulation, PRR-mediated innate immune responses, and local hypoxia in lung tissue (79–81). Fungal infection induces HIF-1α expression through a local hypoxic microenvironment and vascular damage (82, 83). Upregulation of HIF-1α can reduce *Candida albicans* colonization and alleviate airway inflammation caused by Aspergillus infection (84).

The latest research reveals that recombinant thrombomodulin (rTM) exerts a protective effect against sepsis by inhibiting the HIF-1α/METTL3/PFKM axis and promoting the transformation of macrophages to an anti-inflammatory phenotype (85). Studies also find that monocytes with high CD38 expression (CD38high monocytes) enhance the pro-inflammatory response by activating the cAMP-PKA signaling pathway, and their expression level is closely related to disease severity and mortality (86). Targeted inhibition of CD38 can effectively reduce the inflammatory response and improve clinical prognosis (86). Elucidating the seemingly paradoxical role of HIF-1α in sepsis is the core of future research. This requires the construction of cell-specific and time-controllable genetic models to reveal its spatio-temporal regulatory mechanisms that vary with cell types, activation timing, and the nature of pathogens. The ultimate goal is to decipher the interaction network between HIF-1α and immunometabolic reprogramming and develop precise targeting strategies.

#### 4.4.2 mTOR pathway

mTOR, as a key intracellular signal-regulating hub, precisely regulates core cellular activities such as cell growth, proliferation, and autophagy by integrating signals from nutrients, energy, and growth factors, and is an important pharmacological target for autophagy regulation (87). mTOR functions in two functionally different complexes: mTORC1 mainly regulates protein synthesis, cell growth, and inhibits autophagy, while mTORC2 focuses on regulating cell survival and dynamic reorganization of the cytoskeleton (88, 89). These two complexes work together to maintain cellular homeostasis and function.

In sepsis, activation of the mTOR signaling pathway by inflammatory mediators and growth factors promotes protein synthesis and cell proliferation (90). However, excessive activation of this pathway leads to metabolic disorders and functional abnormalities in immune cells (90). Studies show that mTOR can exacerbate pyroptosis of CD4^+^ T cells by negatively regulating the PPARγ-Nrf2 signaling pathway, promoting the occurrence of sepsis-related immune suppression and ultimately resulting in poor clinical prognosis (54). In terms of treatment strategies, Shenfu injection specifically activates the PI3K-AKT signaling pathway, upregulates the expression of autophagy-related proteins, and inhibits cell apoptosis, effectively reducing sepsis-induced lung tissue damage in ARDS and improving lung function (91). Similarly, liensinine regulates the PI3K/AKT/mTOR signaling pathway, significantly inhibits cardiomyocyte apoptosis, and enhances antioxidant defense ability, thereby improving sepsis-related myocardial dysfunction and reducing myocardial damage (92). These results indicate that the mTOR signaling pathway plays a crucial role in sepsis-related multiple organ dysfunction. Its abnormal activation not only affects immune system function but also participates in the damage process of important organs such as the lungs and heart, suggesting that targeted regulation of the mTOR pathway may be a new therapeutic strategy for multi-organ protection in sepsis. However, the clinical translation results of mTOR inhibitors have been disappointing, which may be due to the dual role of mTOR in promoting both beneficial anabolic processes and harmful inflammatory signals. Future research must clarify the precise intervention time window and the patient subgroups (e.g., those with significant mTOR over-activation characteristics) for mTOR inhibitors.

#### 4.4.3 Sirtuins family

Sirtuins are a class of NAD^+^-dependent deacetylases, and there are seven subtypes (SIRT1–7) in mammals (93). Sirtuins play a central regulatory role in cellular processes such as gene expression regulation, chromatin structure maintenance, mitochondrial function, DNA damage repair, and metabolic regulation by mediating acetylation modifications of histones and non – histones (93, 94). Different subtypes have unique sub-cellular localization and functional characteristics: SIRT1 is mainly located in the nucleus and participates in metabolic regulation and inflammatory responses (95, 96); SIRT3 is specifically distributed in mitochondria and regulates mitochondrial function and oxidative stress responses (97, 98). Notably, members of the Sirtuins family maintain cellular redox homeostasis by coordinately regulating key signaling pathways such as NRF2, FOXO, and PGC-1α. SIRT1, SIRT3, and SIRT6 promote mitochondrial biogenesis by deacetylating PGC-1α, jointly forming a precise defense network for cells against oxidative stress (99).

Research shows that the Sirtuins family plays a key immunomodulatory role in different pathological stages of sepsis (100). In the high-inflammation stage, Sirtuins significantly reduce the production of pro-inflammatory cytokines by inhibiting NF-κB activity; in the low-inflammation stage, they promote the immunosuppressive state by deacetylating histones and regulating DNA methylation (100). Meanwhile, Sirtuins enhance the antioxidant defense ability of cells by activating NRF2 (100). Specifically, SIRT1 inhibits the activation of the NLRP3 inflammasome and the STING pathway through a Rab7-dependent late endosome-mitophagy pathway, thereby reducing sepsis-related acute lung injury (101); SIRT3 mediates melatonin-induced deacetylation of TFAM, promotes mitophagy, and improves sepsis-induced acute kidney injury (102). In addition, β-nicotinamide mononucleotide effectively reduces inflammation and oxidative stress responses in the hippocampus of septic mice by activating the NAD^+^/SIRT1 pathway (103). A key unsolved problem in future research is to clarify the functional redundancy and compensatory relationships among different members of the Sirtuins family in sepsis. On this basis, it is also necessary to further analyze the specific regulatory mechanisms of each subtype and carefully evaluate the efficacy of enhancing their activity through NAD^+^ precursors such as NMN at different disease stages to determine their clinical translation potential and provide new ideas for treatment.

## 5 The gut-lung Axis in Sepsis-related lung injury

### 5.1 Sepsis-induced ALI/ARDS: the role of Enterogenic signals

**Figure 1:**
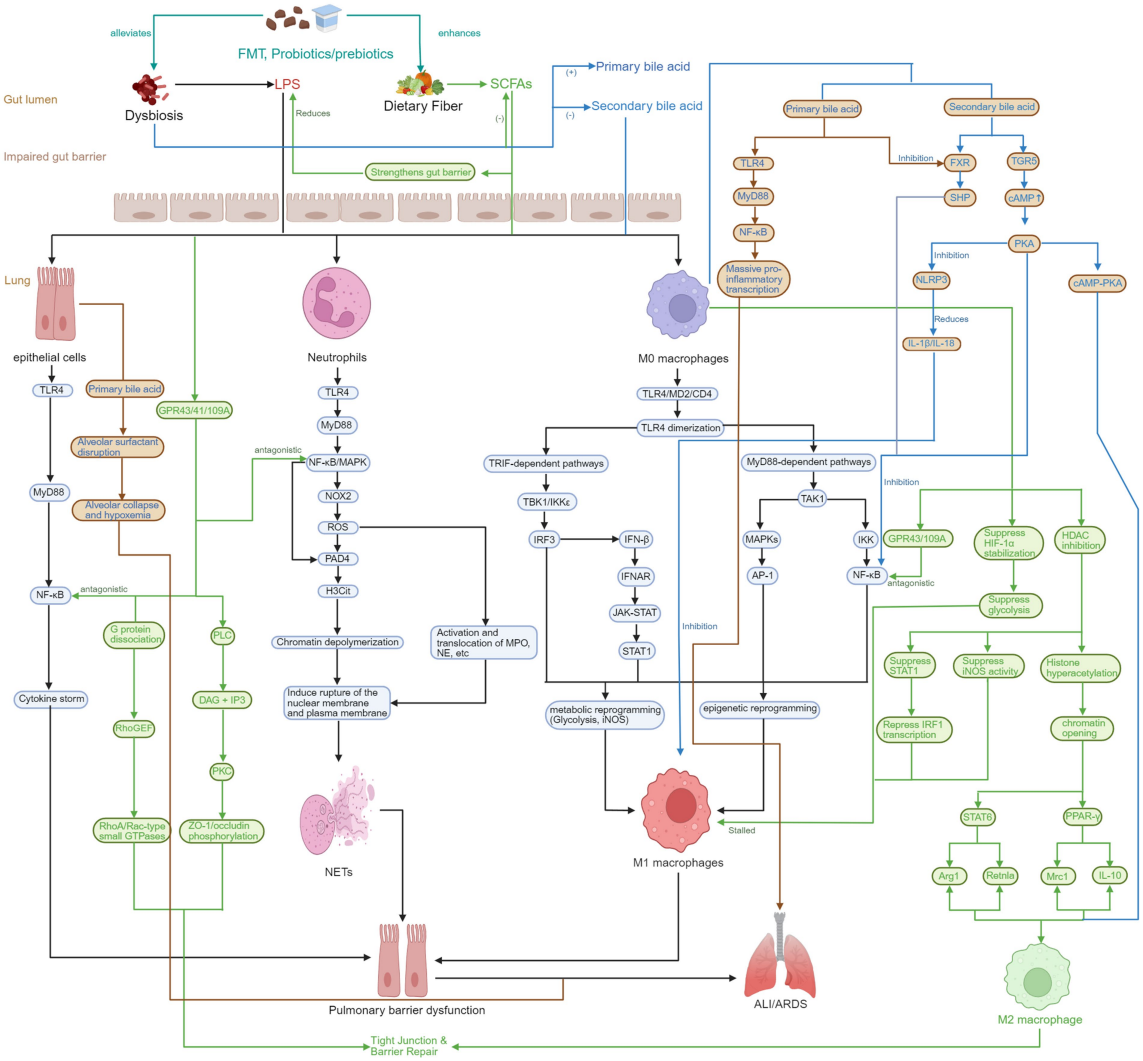
During the pathological process of sepsis, there exists an immunometabolic crosstalk mechanism within the “gut-lung axis.” Specifically, gut microbiota dysbiosis leads to the depletion of short-chain fatty acids (SCFA) and the accumulation of toxins such as lipopolysaccharide (LPS), which in turn results in the disruption of the intestinal barrier. Intestinal-derived LPS can enter the lungs through the blood circulation, activating the TLR4/NF-KB signaling axis of alveolar macrophages. This activation drives the polarization of macrophages towards the M1 phenotype and induces the formation of neutrophil extracellular traps (NETs) by neutrophils ultimately leading to acute lung injury (ALI) or acute respiratory distress syndrome (ARDS). Created with BioRender.com.

In sepsis-induced ALI/ARDS, the imbalance of gut microbiota disrupts the intestinal barrier function, promoting the translocation of bacteria and products such as LPS into the bloodstream. This activates the systemic inflammatory response and exacerbates lung injury (104). Meanwhile, the reduction of beneficial metabolites such as SCFAs (as detailed in Section 3.2) further weakens the mucosal immune defense function, intensifying the activation of oxidative stress and inflammatory signaling pathways, thus forming a vicious cycle in the gut-lung axis and driving the progression of ALI/ARDS (Figure 1) (105). Animal studies have shown that specific strains like *Akkermansia muciniphila* can significantly improve LPS-induced lung injury by enhancing the expression of tight junction proteins, regulating the microbiota structure, and inhibiting the TLR2/MyD88/NF-κB pathway (106). Additionally, microbiota dysbiosis can activate inflammatory pathways in lung tissue, including the TLR4/NF-κB signal (as discussed in detail in Section 6.1), and promote the release of pro-inflammatory factors (107).

In-depth mechanistic studies in mouse models have revealed that sepsis can induce the activation of small intestine-derived CD44^+^ Ly6C^−^ IL-7R^high^ CD8^low^ memory γδT17 cells. These cells migrate to the lung tissue via the circulation and secrete a large amount of IL-17A, exacerbating pulmonary inflammation. Meanwhile, alveolar macrophages form a positive feedback loop through the Wnt/β-catenin/CCL1 axis to promote the infiltration of γδT17 cells into the lungs (108). The *Pseudomonas aeruginosa* infection mice model has confirmed that pulmonary inflammation can inhibit intestinal epithelial proliferation, leading to cell cycle arrest and barrier function impairment, which verifies the bidirectional regulation between the lungs and the intestines (109). Notably, this pattern of immune cell migration is dynamically regulated by the composition of gut microbiota.

In the pathological state of sepsis, specific T-cell subsets such as mucosal-associated invariant T (MAIT) cells and intraepithelial T cells (IEL) are highly likely to be significantly influenced by the gut microbiota. Specifically, derivatives of the gut microbiota, such as riboflavin metabolites and sulfated bile acids, can enter the bloodstream and subsequently activate MAIT cells in the lungs. Once activated, MAIT cells release effector molecules, which not only enhance the body’s antibacterial immune capacity but also strengthen the barrier functions of the intestines and lungs, thereby establishing a “gut-lung” immune axis (110). In parallel, under the stimulation of microbiota signals, IEL can differentiate into a regulatory phenotype. This regulatory-phenotype IEL contributes to maintaining the stability of the intestinal microenvironment and effectively inhibits excessive inflammatory responses (111, 112). In conclusion, these research findings offer new insights for the treatment of sepsis. In particular, they provide a solid theoretical basis and clear research directions for targeted therapeutic strategies focusing on the microbiota-immune interaction.

### 5.2 Gut microbiome and lung macrophage polarization (M1/M2 imbalance)

The gut microbiome is an ecological network composed of trillions of microorganisms, forming a dynamic equilibrium system of interdependence and restriction with the host (113, 114). During sepsis, exogenous factors (such as antibiotics and disorder of the body’s immune function) disrupt this equilibrium (115, 116). By regulating the pattern recognition receptor signaling pathway and immune cell polarization (especially the balance of M1/M2 macrophages), it significantly affects the pulmonary immune microenvironment (117). This indicates that the gut microbiome can participate in the immune regulation process of distal organs through the “gut-lung axis” pathway and plays a key regulatory role in the occurrence and development of pulmonary inflammatory diseases.

As important immune effector cells in the lungs, lung macrophages exhibit dynamic polarization characteristics in sepsis-induced ALI (118). In the early stage of ALI, PAMPs such as LPS promote the polarization of lung macrophages into the M1 phenotype through the TLR4/NF-κB signaling pathway (119). Their overactivation can trigger an inflammatory storm and exacerbate lung tissue damage (120, 121). In the later stage of the disease, M2 macrophages play a key role in promoting inflammation resolution and tissue repair, and their different subtypes show significant functional heterogeneity (122, 123).

Clinical studies have shown that the level of Nrf2 mRNA in the peripheral blood of sepsis patients is negatively correlated with the degree of inflammation. The deficiency of Nrf2 can exacerbate the M1/M2 imbalance and lung injury by affecting autophagy and the NF-κB/PPARγ pathway (124). Studies have also found that the expression of Spry4 in macrophages is positively correlated with the severity of ALI, and its deficiency alleviates lung injury by activating the CaMKK2-AMPK pathway (125). Notably, the origin of exosomes determines the direction of their regulation of macrophage polarization: pathogen-derived exosomes promote M1 polarization, while mesenchymal stem cell (MSC)-derived exosomes induce M2 polarization through specific miRNAs, thus alleviating LPS-induced ALI (125).

The current view has gone beyond the M1/M2 dichotomy, emphasizing that macrophages form a highly plastic and functionally continuous phenotypic spectrum under the regulation of microenvironmental signals (126). SCFAs derived from gut microbiota (such as butyrate) can promote the polarization of alveolar macrophages towards an anti-inflammatory phenotype through epigenetic mechanisms such as inhibiting histone deacetylases (HDACs) and regulating cell metabolism (127, 128). On the other hand, PAMPs such as LPS from the gut induce a pro-inflammatory state through the TLR4/NF-κB pathway (129). In-depth understanding of the regulation of the continuous activation spectrum of macrophages by gut-derived signals can provide new strategies for restoring immune homeostasis in sepsis.

Based on the existing research evidence, future research should focus on clarifying the molecular mechanism by which gut microbial metabolites regulate lung macrophage polarization and developing targeted intervention strategies (such as MSC exosomes or specific miRNA preparations). By precisely regulating the M1/M2 macrophage balance in the “gut-lung axis,” new translational medicine ideas can be provided for the treatment of sepsis-related lung injury.

### 5.3 NETs and lung injury: contributions of the gut microbiota

Neutrophils, as important effector cells of the innate immune system, participate in anti-infective immune defense through mechanisms such as phagocytosis ROS production, and the formation of NETs (130). NETs are reticular structures released by neutrophils, composed of de-condensed chromatin fibers decorated with various antimicrobial proteins and histones on the surface, which have physiological functions such as capturing pathogens, regulating immune responses, and promoting coagulation (131–133). In the pathological process of sepsis, the excessive activation of neutrophils leads to the massive release of NETs: on one hand, they limit the spread of pathogens through physical capture and antibacterial effects (134); on the other hand, excessive NETs can exacerbate microcirculation disorders by damaging endothelial cells and increasing vascular permeability, thereby promoting the occurrence and development of multiple organ dysfunction syndrome (135).

Neutrophils exhibit significant functional heterogeneity during the progression of sepsis and can differentiate into different subsets such as N1, N2, and immunosuppressive Nreg. Among them, the number of the low-density neutrophil subset increases abnormally and shows dysfunction, especially the pro-inflammatory Ly6G^+^CD11b^hi^ population induced by extracellular cold-inducible RNA-binding protein (136). Studies have shown that this type of neutrophils can form NETs, activate the DNA-TLR9 signaling pathway, and then induce endoplasmic reticulum stress and a large amount of ROS production, ultimately leading to the impairment of the intestinal barrier function (137). In recent years, it has been found that signals from the gut can differentially regulate neutrophil heterogeneity in sepsis. Gut microbiota dysbiosis promotes the expansion of endogenous LPS (138), thereby expanding the pro-inflammatory Ly6G^+^CD11b^hi^ neutrophil subset. This subset has enhanced NETs-forming ability and elevated levels of pro-inflammatory factor release, is highly pathogenic, and can induce endothelial damage and multiple organ dysfunction (136). In addition, gut microbiota metabolites such as acetate can inhibit neutrophil apoptosis through the FFAR2/FABP4/endoplasmic reticulum stress axis, promote the inflammatory response, and further exacerbate lung injury (139). Lefrançais et al. found a large amount of NETs formation in both severe bacterial pneumonia and ALI mouse models (140). Further animal experiments showed that the gut microbiota metabolite acetate aggravates lung injury by inhibiting neutrophil apoptosis and promoting the release of inflammatory factors through the FFAR2/FABP4/ER stress axis (139). In addition, programmed death-ligand 1 (PD-L1) inhibits neutrophil apoptosis through the PI3K/AKT pathway, and its deficiency can significantly improve the prognosis of sepsis (42).

Future research should further investigate neutrophil heterogeneity, the interaction mechanisms between gut microbiota and the host, and the specific roles of related signaling pathways in the pathogenesis of sepsis. Potential therapeutic strategies include inhibiting neutrophil NET formation, regulating neutrophil apoptosis pathways, intervening in TLR9-mediated endoplasmic reticulum stress, and targeting gut microbiota and their metabolites. Meanwhile, elucidating the bidirectional communication mechanism of the gut-lung axis in sepsis-associated ALI/ARDS provides new perspectives for clinical management. For instance, gut-derived molecules such as succinate and trimethylamine N-oxide may serve as early warning indicators for “gut-derived lung injury,” while interventions like modulating bile acid metabolism (e.g., using ursodeoxycholic acid/UDCA) or activating the vagus nerve anti-inflammatory pathway (e.g., via percutaneous vagus nerve stimulation) have demonstrated potential for organ protection through the gut-lung axis. Future studies should focus on validating the feasibility of these gut-derived molecules as biomarkers and identifying patient subgroups that may benefit from corresponding targeted therapies, thereby advancing precision medicine in sepsis treatment.

### 5.4 Gut microbiota metabolites and lung immunity

In addition to SCFAs, bile acids (BAs), tryptophan metabolites, and others, as key molecules of gut microbial metabolism, are involved in the regulation of immune modulation and organ damage through multiple pathways in sepsis. Recent studies have found that these metabolites can not only affect the inflammatory response via receptor signaling pathways but also directly interfere with the energy metabolic reprogramming of lung immune cells, thereby regulating their functional states.

#### 5.4.1 Bile acid

Intestinal microbiota can transform primary BAs into secondary ones like deoxycholic acid (DCA) and lithocholic acid (LCA). These secondary BAs activate TGR5 and farnesoid X receptor (FXR) receptors on macrophages, inhibit the NF-κB pro-inflammatory pathway, and promote macrophage differentiation into an anti-inflammatory type, maintaining intestinal and systemic immune homeostasis (141). However, in acute lung injury, BAs act as DAMP and are involved in the ARDS-like pathological process. They activate sPLA2 to disrupt alveolar surface tension, trigger inflammation and cytokine storms via the TGR5/NF-κB pathway, and directly cause mitochondrial damage and cell death, leading to alveolar collapse and hypoxemia (142). In contrast, in radiation-induced pulmonary fibrosis, intestinal microbiota remodeling increases DCA and LCA levels. After reaching the lungs, they activate the FXR-SHP pathway, inhibit epithelial-mesenchymal transition and collagen deposition, and delay pulmonary fibrosis progression (143).

#### 5.4.2 Tryptophan metabolites

Indole-related substances produced by the gut microbiota through tryptophan metabolism activate the aryl hydrocarbon receptor (AhR) in lung macrophages and dendritic cells (144). Experiments on asthmatic mice have shown that hydrogen-rich water can reshape the gut microbiota, increase the number of indole-3-acetic acid (IAA)-producing bacteria such as Bifidobacterium, and thus elevate the IAA level. By activating the AhR signaling pathway, it inhibits airway inflammation, achieving immune regulation of the “gut-lung” axis (145). Besides directly regulating the transcription of immune genes, AhR activation can also inhibit glycolysis and promote immune tolerance (146). Kynurenine, a product of another tryptophan metabolic pathway, is also involved in AhR-related immune regulation (147), which is detailed in Section 6.2.

#### 5.4.3 Succinate

Succinate has become a key metabolic-immune signaling mediator beyond its traditional role. In pathologies like gut microbiota dysbiosis or ischemia, circulating succinate levels rise, activating immune-cell SUCNR1 to drive inflammation (148). Mouse gut ischaemia/reperfusion (I/R) model studies show that I/R-induced gut microbiota disruption raises succinate, which spreads to the lungs. There, it activates alveolar macrophage SUCNR1, causing M1 polarization, metabolic reprogramming, and inflammation-factor release. It also damages alveolar epithelial cell mitochondria and induces apoptosis, worsening lung injury (149). Intervening in succinate signaling or regulating gut microbiota can relieve the injury, highlighting gut-derived succinate’s central role in gut-lung axis-mediated distal organ damage.

#### 5.4.4 Trimethylamine N-oxide

Trimethylamine N-oxide (TMAO) is not only associated with cardiovascular diseases but also has immunomodulatory functions (150). In a LPS-induced model of acute lung injury in male septic mice, the metabolism of choline/L-carnitine by gut microbiota is reprogrammed. This leads to the expansion of trimethylamine (TMA)-producing bacteria (such as Anaerococcus and Prevotella), resulting in increased production of TMAO by flavin-containing monooxygenase 3 in the liver. Circulating TMAO activates the NLRP3 inflammasome and the NF-κB signaling pathway in alveolar macrophages, promoting the release of IL-1β/IL-18 and triggering pyroptosis, ultimately exacerbating the damage to the pulmonary vascular endothelial barrier (151). These results indicate that gut microbiota-derived TMAO is a key inflammatory amplifier that exacerbates septic lung injury.

The impact of gut microbiota metabolites on pulmonary immune cell function hinges on their ability to reprogram cellular metabolism. Although succinate/TMAO and IAA are known to promote and inhibit glycolysis, respectively, thereby influencing macrophage inflammatory phenotypes, the precise mechanisms by which they fine-tune energy metabolism across different lung immune cells remain elusive. Elucidating these mechanisms and developing targeted metabolic interventions will offer novel perspectives for preventing and treating pulmonary diseases.

To systematically summarize the immunomodulatory effects of the aforementioned gut microbiota metabolites in the lung, the major metabolite classes, representative molecules, receptors, cellular targets, and net immunological effects are summarized in Table 1.

**Table 1:** Summary of key gut microbiota-derived metabolites and their immunological roles in the lung.

Metabolite class	Representative metabolite(s)	Primary receptor(s)	Cellular targets in the lung	Net immunological effect in the lung
SCFAs	Butyrate, propionate, acetate	GPR43, GPR109A, HDACs	Alveolar macrophages, epithelial cells, T cells	Anti-inflammatory: polarization of macrophages towards an M2 phenotype, augmentation of Treg-mediated immunosuppression, attenuation of pro-inflammatory cytokine release, and enhancement of lung barrier integrity
Bile acids (primary)	Primary BAs (e.g., CDCA)	FXR, TGR5	Alveolar macrophages, epithelial cells	Pro-inflammatory action: characterized by its role as a DAMP that activates the NLRP3 inflammasome and sPLA2, leading to surfactant disruption and inflammation. In contrast, the anti-inflammatory actions are secondary, facilitated by secondary bile acids
Bile acids (secondary)	DCA, LCA	FXR, TGR5	Macrophages, epithelial cells	Anti-inflammatory: blocks NF-κB pathway, induces anti-inflammatory macrophage phenotype, and mitigates radiation-induced pulmonary fibrosis via FXR-SHP signaling
Tryptophan metabolites	IAA, kynurenine	AhR	Macrophages, dendritic cells	Immunoregulatory: mitigates airway inflammation, fosters immune tolerance, suppresses glycolytic reprogramming in immune cells, and modulates T-cell response balance
Succinate	Succinate	SUCNR1	Alveolar macrophages, alveolar epithelial cells	Pro-inflammatory: drives M1 polarization, provokes metabolic reprogramming, stimulates inflammatory factor release, impairs mitochondrial function, and worsens lung injury
TMAO	TMAO	NLRP3 inflammasome, NF-κB pathway	Alveolar macrophages, vascular endothelial cells	Pro-inflammatory: drives NLRP3 inflammasome and NF-κB activation, propagates pyroptotic cell death (IL-1β/IL-18), and impairs the pulmonary vascular endothelium
Acetate	Acetate	FFAR2	Neutrophils	Pro-inflammatory: blocks neutrophil apoptosis via the FFAR2/FABP4/ER stress axis, propagates inflammatory responses, and worsens acute lung injury
LPS	LPS	TLR4, TLR2	Alveolar macrophages, neutrophils, lung epithelial cells	Pro-inflammatory: drives NF-κB and MAPK signaling, promotes NET release, and impairs the epithelial barrier

SCFAs, short-chain fatty acids; NLRP3, pyrin domain containing protein 3; DCA, deoxycholic acid; LCA, lithocholic acid; IAA, indole-3-acetic acid; AhR: aryl hydrocarbon receptor; TMAO, trimethylamine N-oxide; LPS, lipopolysaccharide.

## 6 Mechanistic linkages in patients with Sepsis

### 6.1 Circulating microbial products and pulmonary TLR activation

In recent years, research on the gut-lung axis has revealed the significant role of circulating microbial products in the occurrence and development of pulmonary diseases. When the intestinal barrier function is impaired, microbial products such as LPS, bacterial DNA, and peptidoglycan can enter the bloodstream and migrate to the lungs, activating local immune responses through TLRs (152–154). This section will focus on the detailed mechanisms of this process, particularly in the lung microenvironment.

High mobility group box 1 (HMGB1) is a nuclear protein (155). When cells are damaged or stimulated, HMGB1 is released from the nucleus into the extracellular space and participates in the pathological process of sepsis as a late inflammatory mediator (155). In the early stage of sepsis, stimuli such as LPS and ROS can trigger the release of HMGB1. After binding to CXCL12, it recruits neutrophils and monocytes to the lungs and intestines through the CXCR4 signaling axis, exacerbating inflammation (156). HMGB1 activates the NF-κB pathway through TLR4/TLR2: in the lungs, it promotes the release of inflammatory factors, leading to acute lung injury; in the intestines, it destroys tight junctions, resulting in bacterial translocation and forming a “gut-lung” interactive inflammatory cycle (156). In addition, the HMGB1-TLR2 signal can upregulate PD-L1 on neutrophils, inducing T cell apoptosis and causing immunosuppression, which may ultimately lead to multiple organ dysfunction (156).

TLR4 is a key receptor for LPS-induced lung injury. Its expression level in endothelial cells directly affects the activation of neutrophils and the intensity of the inflammatory response (157). During the occurrence and development of pulmonary diseases, TLR4 does not function independently but participates in the immune response through a complex network of receptor cooperation and signal cross-regulation (158). In the pathogen recognition stage, TLR4 forms a heterodimer with TLR2, enhancing the ability to recognize PAMPs and DAMPs, and synergistically activating NF-κB and MAPK through the MyD88-dependent signaling pathway to promote the release of inflammatory factors (159–161).

In sepsis, LPS binds to the TLR4-MD2-CD14 receptor on lung cells, activates the MyD88-dependent signaling pathway, promotes the ubiquitination of TRAF6 and activates the TAK1/IKKβ/NF-κB pathway, inducing the release of pro-inflammatory factors such as TNF-α and IL-1β (162, 163). This then activates neutrophils, triggers a burst of ROS, and amplifies the inflammatory response through the NLRP3 inflammasome, ultimately leading to pathological changes in ALI such as alveolar-capillary barrier destruction and pulmonary edema (162, 163). Studies have found that the flavonolignan hydronaphthalenin D (HD) from plants can alleviate LPS-induced lung injury through a dual mechanism: on the one hand, it reduces oxidative stress through the Keap1/Nrf2/HO-1 pathway; on the other hand, it reduces the levels of inflammatory factors by inhibiting the MAPK/NF-κB signal and blocks the activation of the NLRP3 inflammasome (164). Further animal experiments have confirmed that HD can significantly improve pulmonary edema and pathological damage, suggesting that targeting the TLR4 pathway, clearing circulating LPS, and regulating the microbiota-immune axis may become new strategies for the treatment of sepsis (164). In the future, multi-organ protective intervention strategies need to be developed by targeting the TLR4 pathway, clearing circulating LPS, and regulating the microbiota-immune axis to contain the uncontrolled inflammation and organ failure driven by microbial products in sepsis.

### 6.2 Bile acid and tryptophan metabolism: effects on pulmonary inflammation

BAs, as steroid substances synthesized by the liver, not only play a key role in intestinal fat digestion and absorption but recent studies have also revealed that their metabolism is closely related to immune regulation (165, 166). In critically ill patients, impaired intestinal barrier function can affect the enterohepatic circulation of bile acids, leading to abnormal levels of circulating bile acids (167, 168). In sepsis, accumulated bile acids act as DAMPs to activate the NLRP3 inflammasome-IL-1β axis, accompanied by the down-regulation of the FXR, forming a “cholestasis-inflammation-organ injury” vicious cycle (169). In the lungs, BAs may inhibit the inflammatory response through receptor-mediated signaling pathways. Studies have shown that ursodeoxycholic acid can inhibit the dsDNA-cGAMP signal transduction by antagonizing the STING-TBK1-IRF3 pathway, block the assembly of the ZBP1-PANoptosome, and simultaneously inhibit PANoptosis mediated by Caspase-3/8/9, GSDMD, and RIPK3-MLKL, thereby reducing the death of alveolar epithelial cells and macrophages, alleviating the cytokine storm, and ultimately improving lung injury and increasing survival rates (170).

Tryptophan is an essential amino acid, and its metabolic pathways include the kynurenine pathway and the serotonin pathway (171). In the state of sepsis, the reprogramming of tryptophan metabolism is mainly manifested by the activation of the kynurenine pathway, and its metabolites play an immunomodulatory role by inhibiting T-cell activation and proliferation, thereby reducing excessive inflammatory responses (172). Studies have shown that tryptophan metabolites can indirectly regulate the intensity of the pulmonary inflammatory response by competitively depleting tryptophan, reducing the synthesis of neurotransmitters such as 5-hydroxytryptamine, and activating the AhR/kynurenine pathway (173). In a rat model of LPS-induced acute lung injury, tryptophan can effectively reduce oxidative stress by inhibiting the NF-κB/MAPK signal pathway, reducing the expression and release of pro-inflammatory factors such as TNF-α, IL-8, and MIF, and significantly up-regulating the activities of superoxide dismutase and catalase and increasing glutathione reserves (174). In addition, tryptophan can also reduce neutrophil infiltration and myeloperoxidase activity, ultimately alleviating LPS-induced acute lung injury (174). It is worth noting that a technology based on DNA-AgNC nanofluorescent probes can achieve the simultaneous quantification of tryptophan and albumin in 1 μL of plasma within 5 min, providing a dual-marker detection scheme for the bedside rapid diagnosis of sepsis (175).

The latest research shows that bile acids and tryptophan, as key metabolites of the gut microbiota, have significant interactions in their metabolic pathways (176). The tryptophan-cholic acid conjugate Trp-CA mediated by gut commensal bacteria can promote GLP-1 secretion through the Gs-cAMP and β-arrestin-1-ALDOA dual pathways by activating the MRGPRE receptor (177). Metformin intervention experiments have confirmed that by regulating the “gut microbiota-bile acid-tryptophan-kynurenine/indole” metabolic axis, the levels of neurotoxic metabolites can be reduced, while 5-HT and GLP-1 can be increased, thereby improving depression-like behaviors (178). This interactive regulatory network of bile acids-microbiota-tryptophan may provide new targets for the treatment of diseases such as sepsis by influencing the inflammatory response and metabolic homeostasis.

### 6.3 Vagus nerve-mediated cholinergic anti-inflammatory pathway

As the longest cranial nerve in the human body, the vagus nerve binds to the α7 nicotinic acetylcholine receptor (α7nAChR) on the surface of immune cells through acetylcholine, inhibiting the release of inflammatory factors and constituting the cholinergic anti-inflammatory pathway (179, 180). In sepsis, the activity of this pathway decreases, leading to uncontrolled inflammation. Its molecular mechanism involves the vagus nerve activating the α7nAChR of alveolar macrophages and epithelial cells, triggering the JAK2/STAT3 and PI3K/Akt signaling pathways, inhibiting the activation of NF-κB and the NLRP3 inflammasome, thereby reducing the release of pro-inflammatory factors and neutrophil infiltration, protecting the alveolar-capillary barrier, and alleviating lung injury (181). In addition, the vagus nerve-adrenal axis is activated under electrical acupuncture or inflammatory stimulation, prompting adrenal medullary chromaffin cells to convert L-DOPA into dopamine through the DDC enzyme (182). Circulating dopamine activates the Gs-cAMP-PKA pathway by binding to the D1R receptor on immune cells, further inhibiting NF-κB and the NLRP3 inflammasome, reducing the levels of inflammatory factors such as TNF-α and IL-1β, and improving the systemic and pulmonary inflammatory responses (182).

Studies have shown that the anti-inflammatory effect mediated by the vagus nerve is closely related to the regulation of the gut-lung axis. In a burn mouse model, vagus nerve electrical stimulation reduces the sympathetic nerve tension in the intestines, maintains the intestinal mucosal barrier function, reduces the release of mediators such as LPS/HMGB1, and then inhibits the NF-κB/ICAM-1 pathway in alveolar macrophages, alleviating pulmonary pathological damage (183). This pathway also affects the function of immune cells by regulating bile acid and tryptophan metabolism, forming a neuro-immune-metabolic network regulatory mechanism (184, 185). In the future, by integrating AI-driven closed-loop neuromodulation, multi-target drug intervention, and neuro-microbiome precision strategies, the spatiotemporal signal network of α7nAChR and the multi-transmitter synergistic mechanism can be analyzed to achieve individualized neuro-immune treatment for diseases such as sepsis.

To summarize the role of neuro-immune signaling in pulmonary immunity during sepsis, Table 2 details key signaling molecules and their immunological effects.

**Table 2:** Neural and circulating signaling molecules associated with the microbiome.

Signal type	Key molecule/pathway	Origin/trigger	Receptor/target in lung	Net immunological effect in the lung
Neuro-immune pathway	Cholinergic anti-inflammatory pathway (vagus nerve)	Vagus nerve stimulation (mediated by the gut microenvironment)	α7nAChR expression on both alveolar macrophages and epithelial cells	Potently anti-inflammatory: dual inhibition of NF-κB and NLRP3 inflammasome activation, suppression of TNF-α and IL-1β production, and preservation of alveolar-capillary barrier integrity
	Dopamine–Gs-cAMP–PKA axis (functional “vagus–adrenal” link)	Modulation of systemic inflammation or electroacupuncture effects, dictated by the gut milieu	D1R on pulmonary immune cells	Anti-inflammatory: circulating dopamine activates the Gs-cAMP-PKA signaling axis, thereby inhibiting NF-κB and NLRP3 activation and ultimately attenuating lung inflammation
Circulating damage Signal	HMGB1	Passively released from damaged intestinal or extra-intestinal cells	Expression of TLR4 and TLR2 on both endothelial and innate immune cells	Pro-inflammatory: recruitment of neutrophils and monocytes, amplification of the cytokine release, disruption of tight junctions, and exacerbation of the gut-lung inflammatory cycle
	mtDNA	mtDNA release subsequent to cellular damage (potentially secondary to gut-derived insults)	Cytosolic cGAS-STING signaling axis and NLRP3 inflammasome	Pro-inflammatory and interferogenic: induces type-I interferon production via the cGAS-STING pathway and synergizes with the NLRP3 inflammasome to drive IL-1β and IL-18 maturation
	eCIRP	Actively secreted in response to metabolic or inflammatory stress	TLR4/TLR2	Pro-inflammatory: expands the pro-inflammatory Ly6G + CD11b^hi^ low-density neutrophil subset, elicits NET formation, and exacerbates endothelial injury

α7nAChR, α7 nicotinic acetylcholine receptor; D1R, dopamine D1 receptor; HMGB1, high mobility group box 1; mtDNA, mitochondrial DNA; NLRP3, pyrin domain containing protein 3; eCIRP, extracellular cold-inducible RNA-binding protein; NET, neutrophil extracellular trap.

## 7 Therapeutic strategies for the gut-lung axis in sepsis

Building upon the complex mechanisms of gut-lung axis immunometabolic regulation, various microbiome-targeted interventions have been developed and show promise in preclinical models. However, their successful translation into clinical practice requires addressing and overcoming the significant heterogeneity within the critically ill patient population and the unique challenges of the ICU setting. The successful application of the interventions discussed below heavily relies on precise patient stratification, strict control of indications, optimal timing, and optimized delivery methods. The following Table 2 and subsequent discussion will focus on examining the mechanisms, clinical outcomes, and practical challenges associated with these strategies.

### 7.1 Probiotics and prebiotics

Existing studies have shown that probiotics (such as Lactobacillus and Bacillus) and prebiotics (including fructooligosaccharides and inulin) exert multiple biological effects by regulating the balance of the gut microbiota (186). Meta-analysis evidence indicates that in patients undergoing elective gastrointestinal surgery, the perioperative use of probiotics or synbiotics can significantly reduce the incidence of postoperative sepsis, and synbiotics show better clinical efficacy and good safety (187). Another meta-analysis shows that Multi-strain probiotic preparations can repair intestinal epithelial damage in COVID-19 patients, regulate inflammatory and immune pathways, and shorten the duration of symptoms and hospital stay (188). However, there is controversy regarding the effectiveness of probiotics in critically ill patients. Some studies have found that the use of *Lactobacillus rhamnosus* GG not only fails to reduce the incidence of VAP but may also increase the risk of adverse events such as bacteremia (189). A retrospective cohort study based on the HCA Healthcare database, found that among ICU patients with central venous catheters using probiotics, with a significantly increased death risk, especially for powder formulations. Thus, strict indication control is needed for probiotic use in critically ill patients (190). Therefore, strict indication criteria should be followed when using probiotics in critically ill patients.

Mechanistically, fructooligosaccharides can enrich Bifidobacterium in obese mice, activate the cAMP/PKA pathway in intestinal L cells, stimulate GLP-2 secretion, enhance tight-junction protein assembly in the colonic epithelium, improve barrier function, and reduce systemic inflammation (191). However, the results of relevant clinical translation studies are inconsistent. A phase II a phase II trial in ICU sepsis patients showed single inulin intervention was ineffective but safe (192). Another a trial on synbiotic preparations in critically ill ICU patients found it could regulate upper gastrointestinal microbiota without clear clinical benefits (193).

In light of the above research evidence, the application of probiotics in critically ill patients, a special population, must be extremely cautious. Clinical observations have found that in patients with immunosuppression or severely damaged intestinal barriers, the use of probiotics (especially Lactobacillus GG) may increase the risk of bacteremia (194–196). In addition, the heterogeneity among different clinical trials in terms of strain selection, dosage, intervention timing, and patients’ baseline microbial status is the key factor leading to inconsistent efficacy. Therefore, it is not advisable to implement a one-size-fits-all probiotic supplementation strategy in the ICU. Future research should focus on using rapid microbiome detection techniques to identify subgroups of patients with specific dysbiosis (such as Bifidobacterium depletion) and appropriate immune status, implement individualized strain interventions, and strictly monitor safety.

### 7.2 Fecal microbiota transplantation

FMT is a treatment method that reconstructs the balance of the gut microbiota by transplanting the fecal microbiota of healthy donors into the patient’s intestine (197). Studies have shown that FMT has potential application value in sepsis treatment, and its mechanisms mainly include reshaping the gut microbiota, repairing the intestinal barrier function, and regulating the immune response (198). In animal models, FMT restores the gut microbiota-butyrate-GPR43/109A axis, significantly enhances the bactericidal ability of alveolar macrophages, and inhibits the TLR4/NF-κB inflammatory pathway, thereby improving the prognosis of *Klebsiella pneumoniae* lung infection (199). In addition, FMT can block the over-activation of the LPS-TLR4-NF-κB axis and relieve acute lung injury (200). Clinical case reports show that after FMT treatment, a 95-year-old patient with COVID-19 complicated by extensively drug-resistant *Klebsiella pneumoniae* infection had an increased abundance of beneficial gut bacteria (such as Prevotella and Lactobacillus) and a reduced pathogen load, and finally recovered and was discharged from the hospital (200). Notably, the colonization of specific strains (such as Oribacterium sp. GMB0313 and Ruminococcus sp. GMB0270) can block the virus dissemination via the gut-lung axis by activating the IL-12-CXCL9-CD8^+^ T cell antiviral loop (11). However, the application of FMT in sepsis treatment still faces many challenges, including inconsistent donor selection criteria, insufficient evidence of safety and efficacy, and lack of standardization of operation techniques, which require further clinical research for improvement.

### 7.3 Short-chain fatty acids

SCFAs, as detailed in Section 3.2, play a central role in maintaining intestinal barrier function and immune homeostasis through mechanisms such as activating G-protein-coupled receptor signaling pathways, inhibiting HDACs, and regulating inflammasome assembly (201, 202). Animal studies have shown that in the CLP sepsis model, sodium butyrate can significantly reduce lung injury and improve survival by inhibiting HDAC3, expanding Treg cells, and enhancing the gut-lung barrier (203); while in the antibiotic-induced gut microbiota dysbiosis mouse model, supplementing SCFAs can inhibit NLRP3/GSDMD-mediated pyroptosis and improve the prognosis of sepsis (204). Clinical analysis has found that in sepsis patients, the reduction of butyrate-producing bacteria (such as Faecalibacterium and Roseburia) and the depletion of butyrate/propionate occur early, and the levels of these metabolites can independently predict 28-day mortality, providing a basis for risk stratification and microecological intervention (such as butyrate precursors or probiotics).

Although pre-clinical studies have accumulated sufficient evidence, the clinical translation of SCFAs in sepsis treatment is still in its infancy (205). The core challenge it faces is the lack of a safe and effective delivery strategy. Direct supplementation of SCFAs has obvious pharmacokinetic drawbacks: when administered orally, SCFAs are easily and rapidly absorbed in the upper gastrointestinal tract, making it difficult to effectively reach the colonic target area; when administered intravenously, systemic exposure may lead to adverse reactions such as acidosis (206). Currently, more feasible approaches include nutritional interventions using specific dietary fibers (such as inulin and resistant starch) to directionally enrich SCFA-producing bacteria, or developing colon-targeted delivery systems (such as pH-dependent or microbial enzyme-triggered capsules) to improve the local bioavailability of SCFAs at the lesion site (207, 208). Future clinical trials need to focus on evaluating the impact of these strategies on pharmacodynamic biomarkers (such as the dynamic changes in fecal SCFAs) and clarifying the association between them and clinical hard endpoints (such as the number of days without ventilator support) to promote the precise application of SCFAs in sepsis treatment.

### 7.4 Dietary fiber dietary intervention

Dietary fiber is a non-digestible carbohydrate polymer with scientifically proven health benefits like improving bowel movement, regulating blood sugar, and grams, yet there is controversy (209).

It plays a crucial role in regulating gut microbiota and influencing infection and inflammation outcomes. Animal studies show that non-fermentable fibers (e.g., cellulose) improve survival in septic mice, reduce inflammation, and mitigate tissue damage by enriching beneficial bacteria and enhancing intestinal barrier function (210). This effect depends on gut microbiota, as it disappears after antibiotic treatment (210). Furthermore, through integrated human and mouse studies and multi-omics analyses, Hecht et al. found that a fiber-free diet promotes pathogen growth, while a high-fiber diet reduces colonization risk through butyrate production and other mechanisms, with *in vitro* experiments revealing the carbon source competition mechanism (211).

A review that included eight studies showed that for critically ill patients with stable hemodynamics, enteral nutrition containing soluble dietary fiber can significantly reduce the incidence of diarrhea, improve the gut microbiota, enhance intestinal barrier function, and has good overall safety (212). However, insoluble fiber is not recommended, especially for patients with intestinal ischemia or severe motility disorders (212). In addition, a retrospective study based on a clinical database found that in septic patients on mechanical ventilation, early intake of a moderate-dose of dietary fiber (3–35% of actual energy intake) is associated with reduced 28-day mortality, showing a J-shaped dose-response relationship, and can shorten ICU stay and ventilation duration (215).

In conclusion, microecological intervention strategies such as probiotics, prebiotics, FMT, SCFAs, and dietary fiber exert multi-level effects in the prevention and treatment of sepsis by regulating the microbiota-immunity-barrier axis. However, the efficacy shows significant individual differences and scenario dependence. For example, the use of probiotics in immunosuppressed patients may increase the risk of infection (213), and the safety of FMT in immunocompromised populations has not been fully evaluated (214). Moreover, differences in strain selection, dosage, administration timing, and patients’ baseline microecological status among different clinical trials are important factors leading to inconsistent results. Therefore, in the future, the “one-size-fits-all” intervention strategy should be abandoned in favor of precision intervention. By integrating metagenomics and artificial intelligence technologies, a multi-dimensional prediction model of “host-microbe-intervention scenario” can be constructed to identify the subgroups most likely to benefit from the intervention. Ultimately, on the basis of thoroughly elucidating the mechanism of action, combined with real-time monitoring and intelligent algorithms, microecological intervention can be promoted to shift from the traditional “empirical microbial supplementation” to the “precision microbiome editing” paradigm (Table 3).

**Table 3:** Microbiome-centric interventions: mechanisms, outcomes, and challenges.

Intervention type	Primary mechanisms	Clinical outcomes	Drawbacks
Probiotics/prebiotics	Modulate microbiota; enhance barrier integrity; attenuate systemic inflammation	Reduce postoperative sepsis; modulate immunity; accelerate convalescence; attenuate inflammation	Bacteremia risk; unclear clinical efficacy; lacks precise strategy
FMT	Restore the microbiota-metabolism axis, reinforce the mucosal barrier, suppress inflammation, and enhance immune homeostasis	Combat pulmonary infection, prevent the colonization of drug-resistant pathogens, and enhance innate immune defenses	The mechanism of action is ill-defined; clinical efficacy is contentious, there is a potential risk of iatrogenic infection, and it lacks support from randomized controlled trials (RCTs)
SCFAs	Epigenetic orchestration of immune responses, maintenance of immune microenvironment homeostasis, and curtailment of excessive inflammation	Pulmonary protection and barrier restoration, immunomodulation, and basis for risk stratification	Lacks clinical validation, the administration route and dosage require optimization, and the safety and efficacy profiles of some metabolites are not fully established
Dietary fiber dietary intervention	Promote the proliferation of beneficial bacteria, enhance intestinal barrier function, and antagonize pathogenic bacteria	Enhance survival and attenuate systemic inflammation	Lack of standardization in fiber type and dosage, and significant heterogeneity in individual tolerance and efficacy

FMT, fecal microbiota transplantation; SCFAs, short-chain fatty acids.

## 8 Discussion

This review systematically elaborates on the bidirectional immunometabolic regulatory mechanism of the gut-lung axis in sepsis and its crucial role in sepsis-related organ dysfunction. The analysis indicates that gut microbiota dysbiosis is not only a consequence of sepsis but also a key amplifier driving its pathological process. It disrupts the intestinal barrier, reduces the production of beneficial metabolites such as SCFAs, and promotes endotoxin translocation. Subsequently, it remotely activates alveolar macrophages, promotes the formation of neutrophil extracellular traps, and disrupts the balance of regulatory T cells/Th17 cells, ultimately exacerbating lung injury. Conversely, pulmonary inflammation can aggravate intestinal injury through circulating mediators, forming a vicious cycle of the “gut-lung axis,” which provides a new perspective for understanding sepsis-related multiple organ dysfunction.

From a molecular mechanism perspective, this review elucidates a network of immune cell dysfunction predominantly governed by metabolic reprogramming. Key signaling pathways—including HIF-1α, mTOR, in various stages of sepsis, relevant pathways play core and interrelated roles. As detailed in Section 4, these pathways do not operate independently but form a dynamic and often mutually antagonistic network. Our in-depth analysis reveals that during the hyperinflammatory phase, the stabilization of HIF-1α and the activation of mTOR drive the metabolic shift towards glycolysis, promoting the cytokine storm. Conversely, the subsequent decrease in Sirtuins activity and the occurrence of mitochondrial dysfunction mark the transition to the immunosuppressive phase. Notably, the effects of these pathways are not static. For example, the continuous signaling of mTOR exacerbates T-cell exhaustion, while HIF-1α may have tissue-protective functions under specific conditions. The nuances and controversies discussed in Sections 4.2.1 and 4.4 highlight the limitations of the binary view and emphasize the necessity of understanding these mechanisms from a spatio-temporal and contextual perspective.

Although most current mechanism studies are derived from pre-clinical models, they together reveal the underlying causes of the clinical heterogeneity of sepsis: there are significant differences in the dynamic changes of key pathways such as gut microbiota, immune and metabolic responses among different patients. This molecular and cellular heterogeneity, combined with the dynamic nature of core pathways, is the fundamental reason for the repeated failures of “one-size-fits-all” treatment methods. Therefore, the key direction for the future is to translate the above-mentioned mechanistic insights into biomarkers for patient stratification, so as to identify the specific immunometabolic states of patients (e.g., “HIF-1α-dominated hyperinflammatory type” or “Sirtuins-deficient immunosuppressive type”). The phenomena of short-chain fatty acid depletion, dysbiosis, and low expression of monocyte HLA-DR mentioned in the text are the external manifestations of the underlying specific pathway disorders, which together lay the foundation for constructing the immunometabolic classification theory of sepsis.

Current microbiome-targeted intervention strategies show potential in pre-clinical studies, but their clinical efficacy is highly heterogeneous. This reality highlights the necessity of patient typing. The analysis reveals that the success or failure of these interventions depends on four key factors: patient selection, timing of intervention, delivery method, and verification of the targeted biological effects. When applying probiotics or fecal microbiota transplantation in immunosuppressed patients, the risks of bacteremia or iatrogenic infections must be strictly evaluated; the effects of SCFA supplementation at different stages of the disease may be completely opposite; systemic administration of SCFAs faces pharmacokinetic challenges, while targeted delivery to the colon or the use of dietary fiber precursors is a more feasible strategy. Therefore, simply listing intervention measures is no longer meaningful. The core lies in selecting the right intervention for the right patient at the right time.

**Figure 2:**
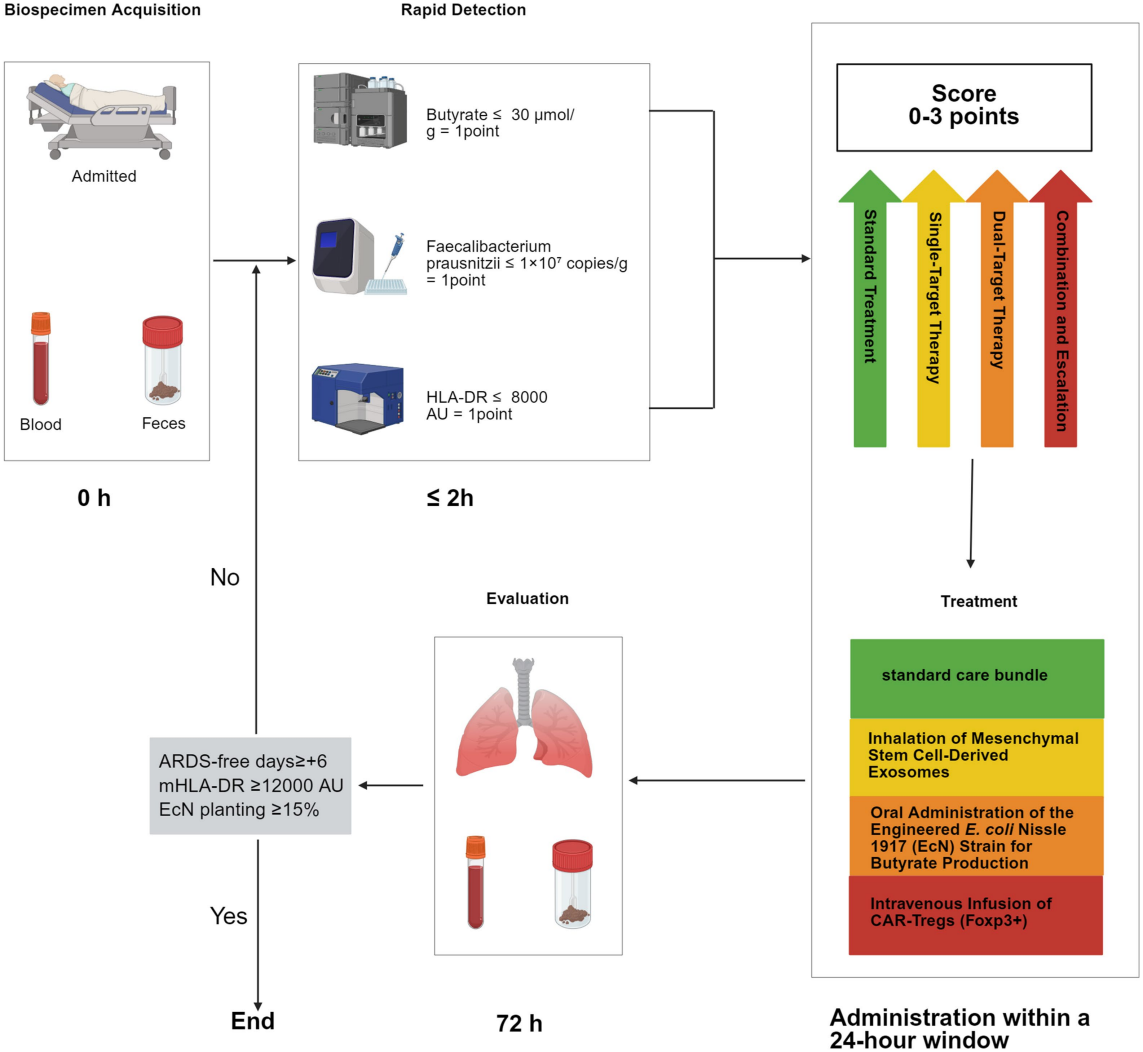
This flowchart depicts the precision treatment pathway for sepsis based on the gut-lung axis. After admission, rapid tests for fecal butyric acid, *Faecalibacterium prausnitzii* abundance, and monocyte HLA-DR levels are performed for scoring (0–3 points). Patients are stratified according to scores and matched with treatment regimens such as standard care, inhalation of mesenchymal stem cell exosomes, oral intake of butyric-acid-producing engineered bacteria, and CAR-Tregs infusion. Treatment starts within 24 h and efficacy is evaluated 72 h later for adjustment, reflecting the precision medicine strategy from biomarker typing to targeted intervention. Created with BioRender.com.

Based on the above discussion, this paper proposes a translational roadmap from the current understanding to future clinical practice, with the core shift from “population-based medicine” to “precision microbiome and immunometabolic editing.” Firstly, a multi-dimensional biomarker typing system should be constructed. It is recommended to establish a rapid typing system integrating microbiological, metabolic, and immunological dimensions at the bedside. Specifically, it can be operated through a simple score to evaluate the level of *Faecalibacterium prausnitzii* in feces, fecal butyric acid concentration, and the expression of human leukocyte antigen-DR on monocytes. According to the typing results, patients can be directed to the most beneficial treatment pathways (Figure 2).

This static stratification framework can be further upgraded to a dynamic and adaptive treatment system in the future. By using clinical data and multi-omics indicators at admission and 72 h, a machine-learning model can be trained to dynamically predict the risk of patients developing acute respiratory distress syndrome and the probability of response to specific interventions. These prediction results can be pushed to doctors in real-time through the clinical information system, enabling dynamic re-stratification and individualized treatment adjustment during the treatment process, forming a closed-loop precision medicine system. This represents a new direction of synergistic treatment combining neuroimmune regulation and microbiome intervention.

In summary, the gut-lung axis plays a central role in the pathogenesis of sepsis, and regulatory strategies based on the microbiome and immunometabolism hold great promise. However, many challenges still need to be overcome to establish a complete evidence chain from mechanistic research to clinical application. Future research should focus on constructing and validating a multi-omics dynamic biomarker system, conducting biomarker-guided prospective randomized controlled trials to optimize intervention strategies, and exploring in-depth the synergistic effects of neuroimmune regulation and microbiome intervention. Through multi-disciplinary collaboration and large-scale precision clinical trials, it is expected to finally achieve the complete translation from basic discoveries to clinical practice, completely revolutionize the treatment pattern of sepsis, and improve patient prognosis.

## 9 Conclusion

This study conducts an in-depth analysis of the bidirectional regulatory mechanism of the gut-lung axis in sepsis. It highlights the pathological processes through which gut microbiota dysbiosis exacerbates systemic inflammation and lung injury via multiple pathways, including alterations in metabolites (such as bile acids and tryptophan), inhibition of the anti-inflammatory pathway mediated by the vagus nerve, and activation of TLR signals in the lungs by circulating toxins. The article systematically reviews the synergistic effects of key pathways, such as HIF-1, mTOR, and sirtuins, in the immunometabolic reprogramming of sepsis. Moreover, it emphasizes the central role of mitochondrial dysfunction and oxidative stress in multiple organ failure. Although microbial intervention strategies have shown certain potential in basic research, their clinical translation still faces challenges such as heterogeneity in efficacy and safety. In the future, it is necessary to develop a dynamic typing system guided by multi-omics and combine neuroimmune regulation with microbial targeted therapy to promote the establishment of a new model for personalized treatment of sepsis.
